# Anti-Müllerian Hormone Is Not Associated with Cardiometabolic Risk Factors in Adolescent Females

**DOI:** 10.1371/journal.pone.0064510

**Published:** 2013-05-31

**Authors:** Emma L. Anderson, Abigail Fraser, William McNally, Naveed Sattar, Hany Lashen, Richard Fleming, Scott M. Nelson, Debbie A. Lawlor

**Affiliations:** 1 MRC CAiTE Centre, University of Bristol, Oakfield House, Oakfield Grove, Bristol, United Kingdom; 2 School of Medicine, University of Glasgow, Glasgow, United Kingdom; 3 BHF Glasgow Cardiovascular Research Centre, University of Glasgow, Glasgow, United Kingdom; 4 Section of Reproductive and Developmental Medicine, University of Sheffield, Sheffield, United Kingdom; University of Padova, Italy

## Abstract

**Objectives:**

Epidemiological evidence for associations of Anti-Müllerian hormone (AMH) with cardiometabolic risk factors is lacking. Existing evidence comes from small studies in select adult populations, and findings are conflicting. We aimed to assess whether AMH is associated with cardiometabolic risk factors in a general population of adolescent females.

**Methods:**

AMH, fasting insulin, glucose, HDLc, LDLc, triglycerides and C-reactive protein (CRP) were measured at a mean age 15.5 years in 1,308 female participants in the Avon Longitudinal Study of Parents and Children (ALSPAC). Multivariable linear regression was used to examine associations of AMH with these cardiometabolic outcomes.

**Results:**

AMH values ranged from 0.16–35.84 ng/ml and median AMH was 3.57 ng/ml (IQR: 2.41, 5.49). For females classified as post-pubertal (n = 848) at the time of assessment median (IQR) AMH was 3.81 ng/ml (2.55, 5.82) compared with 3.25 ng/ml (2.23, 5.05) in those classed as early pubertal (n = 460, P≤0.001). After adjusting for birth weight, gestational age, pubertal stage, age, ethnicity, socioeconomic position, adiposity and use of hormonal contraceptives, there were no associations with any of the cardiometabolic outcomes. For example fasting insulin changed by 0% per doubling of AMH (95%CI: −3%,+2%) p  = 0.70, with identical results if HOMA-IR was used. Results were similar after additional adjustment for smoking, physical activity and age at menarche, after exclusion of 3% of females with the highest AMH values, after excluding those that had not started menarche and after excluding those using hormonal contraceptives.

**Conclusion:**

Our results suggest that in healthy adolescent females, AMH is not associated with cardiometabolic risk factors.

## Introduction

Anti-Müllerian hormone (AMH) is increasingly recognised as a biomarker of the ovarian reserve in adults, due to its strong correlation with primordial follicle number, [1] follicular recruitment rates, [2] response to exogenous gonadotrophins [2] and ability to predict the duration of the reproductive lifespan. [3] In children, similar associations between AMH and the ovarian reserve have been observed. Specifically, girls with a reduced ovarian reserve and shorter reproductive lifespan due to Turners syndrome have low AMH levels, [4] in prepubertal and peripubertal girls AMH levels reflects follicular recruitment rates, [2], [5] and as observed in adults, AMH is negatively associated with follicle stimulating hormone (FSH) in girls from 5 to 15 years. [6] Collectively these studies suggest that AMH may be a useful surrogate of the ovarian reserve and follicular recruitment throughout life.

Premature ovarian insufficiency has been associated with abnormal cardiovascular risk factors and increased cardiovascular mortality [7]–[9]. Ovarian decline and its sequelae are however increasingly being viewed as a continuum rather than an abrupt event at the time of menopause. Investigation of the nature of the relationship between ovarian aging and cardiovascular risk in premenopausal women, has been more limited, but has demonstrated that atherosclerotic lesions predate the onset of the menopause, [10] and that women with reduced ovarian reserve as detected by an elevated FSH but normal oestrogen concentrations exhibit abnormal lipid profiles. [11] The stronger relationship between AMH and ovarian reserve than that observed of the latter with FSH, [1] has consequently led to AMH being examined in adults relative to established cardiometabolic risk factors, including lipids, insulin, C-reactive protein (CRP) and body mass index (BMI),[12]–[23] and in animal models, with atherosclerosis plaque size. [24] These studies have produced conflicting results; for example the association of AMH with insulin has been reported as being positive, [20], [25] negative, [26], [27] or null. [28] This discordance may potentially reflect the relatively small sample sizes studied and the use of select populations, such as women with PCOS. [20], [25], [27] Furthermore, there may be confounding by factors such as smoking and socioeconomic status, [29], [30] or bias due to reverse causality with compromised ovarian vascular function adversely impacting on AMH concentrations. [31].

It would therefore be valuable to study the associations of AMH with cardiometabolic factors in a large, well phenotyped cohort of women from the general population and without established disease. Doing this in a young (adolescent) age group would be valuable because at this age associations are less likely to be confounded by behavioural lifestyle characteristics such as smoking or subject to bias, for example by reverse causality, as atherosclerosis at this age is rare. Furthermore, both mathematical modelling of cross-sectional data and prospective longitudinal data have suggested that AMH is relatively stable at this age, [4], [32], [33] and importantly these cardiometabolic risk factors in adolescence are indicators of future cardiovascular risk. In postmortem studies they have been related to extent of clinically relevant artherosclerosis. [34] Blood pressure in adolescence/early adulthood has been shown to relate to future cardiovascular disease mortality with similar magnitudes of association to those seen for blood pressure in middle age, [35] and blood pressure, markers of dysglycaemia and dyslipidaemia in childhood/adolescence are associated with carotid intima media thickness in mid-adulthood with similar magnitudes of association to the same risk factors measured at the same time as carotid intima media thickness. [36], [37].

The aim of this paper is to examine associations of AMH with fasting insulin, glucose, lipids and CRP in a general population of females with a mean age of 15.5 years.

## Subjects and Methods

Ethical approval for the study was obtained from the Avon Longitudinal Study of Parents and Children Law and Ethics Committee (IRB# 00003312) and the Local Research Ethics Committees (Bristol and Weston, Southmead, and Frenchay Health Authorities). Written informed consent was obtained from all participants in the study. Parents provided written informed consent for their child.

### Study Population

**Figure 1:**
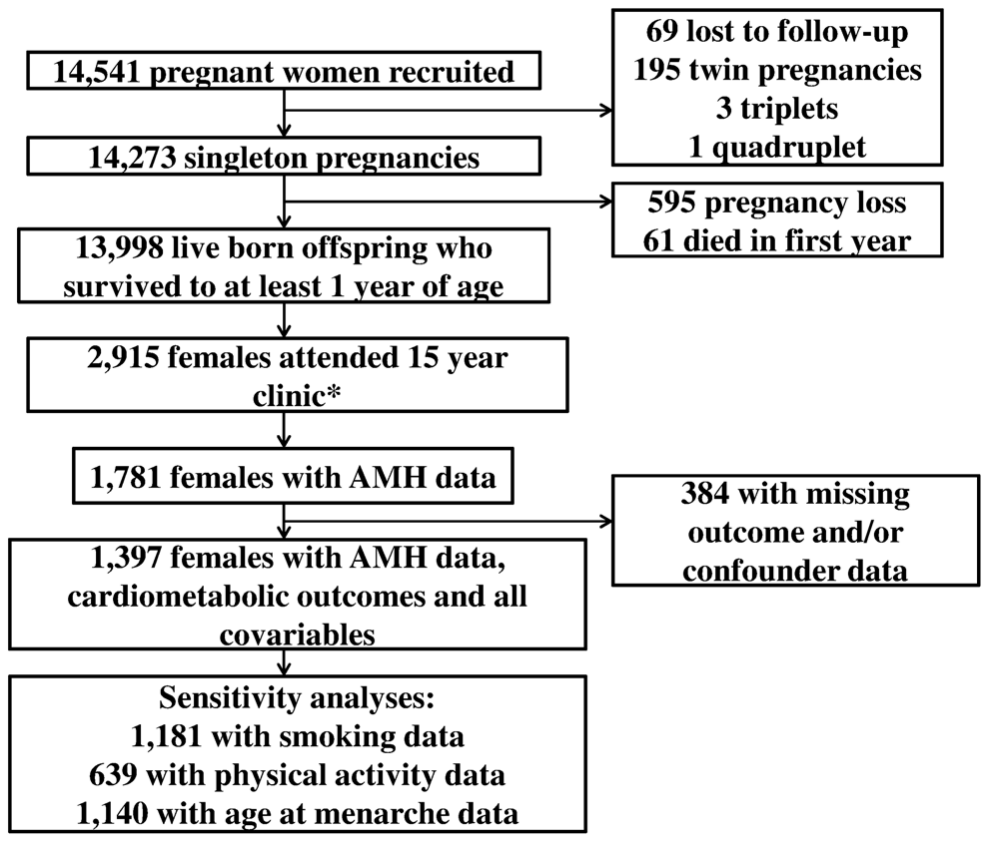
Participant flow through the study. *Participants who had withdrawn, were lost to follow-up or had died were not invited.

The Avon Longitudinal Study of Parents and Children is a population-based, prospective birth cohort, investigating factors that affect the health and development of children. Detailed methods of ALSPAC have been described previously, [38], [39] and are on the study website (www.alspac.bris.ac.uk). Briefly, 14,541 pregnant women resident in the Bristol area with an expected date of delivery between 1st April 1991 and 31st December 1992 were enrolled into the cohort, and of these, 13,988 had a live-born child who was still alive at age 1 year. Participants who attended the 15 year follow-up clinic and who had data on AMH were eligible for inclusion in our study (n = 1,781). Our study sample consists of 1,308 female adolescents (13 sets of twins) who had complete data on AMH, cardiometabolic outcomes and all potential confounders (see **Figure 1**). Ethical approval was obtained from the ALSPAC Law and Ethics committee and relevant local ethics committees in line with the Declaration of Helsinki, and written informed consent was provided by all participants.

### Assessment of Serum AMH

For all blood measures, participants were asked to fast overnight if attending the morning clinic, or for a minimum of 6 hours if attending after lunch. All blood samples were immediately spun and frozen at −80°C. Assays were completed on serum using the commercial AMH generation II ELISA kit provided by Beckman Coulter (Beckman Coulter UK Ltd, High Wycombe, UK). [40] Inter and intra-assay CVs were both <5%.

### Assessment of Outcomes

Serum insulin was measured with an enzyme linked immunosorbent assay (ELISA, Mercodia, Uppsala, Sweden) that does not cross-react with proinsulin. Plasma glucose and CRP were measured with an automated assay, the latter with excellent sensitivity down to 0.1 mg/l. Plasma triglyceride, total cholesterol and HDL-c concentrations were measured by modification of the standard Lipid Research Clinics Protocol by using enzymatic reagents for lipid determination, and LDL-c concentration was determined from these, using the Friedwald equation. [41] Inter and intra-assay CVs for lipids, glucose and CRP were all <5%. For insulin, inter-assay CV is <9.3% and intra-assay CV were <6.0%. It has been established in children and adults that fasting insulin is very highly correlated with formulae that attempt to more accurately measure insulin resistance, such as HOMA-IR or QUICKI, by combining fasting glucose and insulin. [42], [43] A recent consensus statement recommends that fasting insulin be used (rather than HOMA-IR or QUICKI) in epidemiological studies of children/adolescents. [44] In our sample fasting insulin was very highly correlated with HOMA-IR (Pearson’s correlation coefficient 0.98) and QUICKI (0.97) and all results were identical if either of these were used in place of fasting insulin. We therefore present only results with fasting insulin.

### Assessment of Potential Confounders

The following were considered as potential confounders: birth weight, gestational age, pubertal stage, age, age at menarche, ethnicity, socioeconomic position, adiposity, level of physical activity, smoking status and the use of hormonal contraceptives. Birth weight was recorded in the delivery room and abstracted from obstetric records and/or birth notifications. Gestational age was abstracted from clinical records. Pubertal staging was assessed by annual questionnaires which were mailed to participants from age 8 to age 17. The puberty questionnaire could be answered by the participant, a parent, a guardian, or any combination of these individuals. On each questionnaire, the respondent was asked to examine line drawings representing the five Tanner stages for breast size and for pubic hair and to record which drawing most nearly represented the participant’s current stage of development. [45], [46] In each questionnaire, the participant was also asked whether she had had her first menstrual period and, if so, the month and year in which it occurred. Pubertal stage at the time of AMH measurement was established by taking the highest self-reported Tanner rating for either breast development or pubic hair, and using whichever was available if one of these was missing. [45] Whether the participant had started menstruating and if so, at what age this occurred was reported by the participant or her mother in questionnaires administered repeatedly between age 97 and 192 months. The small number of participants who had still not started menstruating at the time of the last questionnaire (N  = 6 (0.46%)) were allocated an age of menarche of 204 months (17 years) in order to allow all participants to be included in analyses. Age was recorded at the time of clinic assessment. Ethnicity was based on the mother’s and her partner’s ethnicity as reported by the mother. Fat mass was measured using a Lunar Prodigy Dual-energy X-ray absorptiometry (DXA) scanner (GE Medical Systems Lunar, Madison, WI). Scans were visually inspected and realigned where necessary. Height was measured to the nearest 0.1 cm with a Harpenden stadiometer with the participant unshod. To assess physical activity, all participants attending the clinic were asked to wear an Actigraph accelerometer (Actigraph LLC, Fort Walton Beach, Florida) for seven days. Data from participants who had worn the Actigraph for at least ten hours per day for at least three days were considered valid. [47] Two main physical activity variables were derived; average accelerometer counts per minute (CPM) over the full period of valid recording, and average number of minutes in moderate to vigorous physical activity (MVPA), per valid measurement day. [48] Based on questionnaire responses, the highest parental occupation was used to allocate participants to family social class groups using the 1991 British Office of Population and Census Statistics (OPCS) classification. Participants were asked about their smoking habits by questionnaire at mean age 14.3 years (the questionnaire administered closest to, and before, the time of the assessment where blood samples were taken) and these data were used to generate a three-level categorical smoking variable: ‘none’ (including those who have never tried a cigarette and those who used to smoke sometimes, but never smoke now), ‘<1 cigarette per week’ or ‘>1 cigarette per week’. Information on use of the hormonal contraceptive pill in the last year was asked in a single question, administered at the same time as the assessment where blood samples were taken. This question was answered by 1,283 (98%) of our study sample. Of the 25 study participants who did not answer this question, 13 indicated in a separate question concerned with recent sexual activity that they were taking the pill, and a further 12 who did not answer either of these questions had an assay result for sex hormone binding globulin (SHBG) >100 nmol/l, which was considered to indicate use of hormonal contraceptives.

### Statistical Analysis

All analyses were conducted in Stata/MP 11.2 (StataCorp, College Station, Texas). AMH, fasting insulin, triglycerides and CRP were positively skewed. In descriptive analyses medians and interquartile ranges (IQR) are presented for these variables, and their natural logs were used in regression models; residuals in these models were approximately normally distributed after these transformations. Consistent with the age of assessment, there were no participants in Tanner stages 1 and 2, and few participants in Tanner stage 3 (**Table 1**). Therefore we generated a binary variable categorising Tanner stage 5 as post-pubertal and stages 1–4 as early pubertal.

**Table 1:** Characteristics of participants included in the study (n = 1,308), and excluded due to missing data (n = 473).

	Included participants (total n = 1,308)		Excluded participants**(total n = 473)		P
	N with available data	Distribution	N with available data***	Distribution	
Median AMH (ng/ml) (IQR)	1,308	3.57 (2.41, 5.49)	473	3.77 (2.60, 5.62)	0.13*
**Outcomes**					
Median fasting insulin-iu/l (IQR)	1,308	9.72 (7.39, 12.79)	471	10.31 (7.51, 14.08)	<0.01*
Mean glucose - mmol/l (SD)	1,308	5.12 (0.34)	471	5.18 (0.49)	<0.01
Mean HDLc - mmol/l (SD)	1,308	1.36 (0.30)	471	1.33 (0.29)	0.11
Mean LDLc - mmol/l (SD)	1,308	2.16 (0.56)	471	2.23 (0.62)	0.04
Median triglycerides - mmol/l (IQR)	1,308	0.76 (0.62, 0.98)	471	0.79 (0.62, 1.04)	0.11*
Median CRP - mg/l (IQR)	1,308	0.39 (0.22, 0.86)	471	0.43 (0.22, 1.07)	0.08*
**Potential confounders**					
Mean birth weight in kg (SD)	1,308	3.38 (0.48)	355	3.37 (0.57)	0.80
Mean gestational age in weeks, (SD)	1,308	39.60 (1.66)	386	39.43 (1.93)	0.10
Mean z-score of fat mass	1,308	0.02 (0.96)	351	0.07 (1.08)	0.46
Non-white (%)	1,308	45 (3.44)	294	21 (7.14)	<0.01
Non-manual social class (%)	1,308	1,127 (86.16)	473	413 (87.32)	0.53
Mean age in years (SD)	1,308	15.41 (0.27)	468	15.65 (0.50)	<0.001
Taking hormonal contraceptives (%)	1,308	170 (13%)	304	79 (26%)	<0.001
Body mass index category (%)					
Underweight	1,308	82 (6.27)	439	30 (6.83)	0.29
Normal weight		957 (73.17)		305 (69.48)	
Overweight		212 (16.21)		76 (17.31)	
Obese		57 (4.36)		28 (6.38)	
Pubertal stage**** (%)					
Stage 1	1,308	0	350	1 (0.29)	0.04
Stage 2		0		1 (0.29)	
Stage 3		24 (1.83)		10 (2.86)	
Stage 4		436 (33.33)		124 (35.43)	
Stage 5		848 (64.83)		214 (61.14)	
**Covariables additionally adjusted for in the sensitivity analyses**					
Median CPM (IQR)	607	420.58 (339.33, 511.12)	142	425.60 (350.32, 523.85)	0.66*
Median MVPA (IQR)	607	15.14 (7.57, 27.00)	142	15.55 (8.66, 29.71)	0.88*
Ever smoked (%)	1,096	336 (30.57)	305	95 (31.15)	0.85
Median age at menarche in years (IQR)	1,191	12.83 (11.83, 13.67)	335	13.00 (11.75, 13.83)	<0.01*

IQR – interquartile range. SD – standard deviation.

For continuous variables the difference between the means of those included and excluded from the analysis was tested using an unpaired t-test.

For categorical variables the difference between those included and excluded from the analysis was tested using Pearson’s chi-squared test.

* For non-normally distributed variables, differences between medians of those included and excluded from the analysis were tested using a Mann-Whitney U-test.

** Excluded participants are those that were eligible for inclusion in our study (i.e. they attended the 15 year and had data on AMH), but who were missing data for cardiometabolic outcomes and/or potential confounders.

*** The ‘N with available data’ for the excluded participant’s column relates to the number of excluded participants that had data for each of the variables included in our analysis. Thus, the means, medians and percentages reported for the excluded participants are based only on those that had data available for each variable.

**** The highest pubertal stage was established by taking the highest Tanner rating for either breast development or pubic hair. If there were missing data for breast development, pubic hair ratings were used where available and vice versa

In order to examine whether there were non-linear associations of AMH with outcomes and to ensure correct modelling of potential confounding factors, distributions of outcomes and confounders were examined by quintiles of AMH and tests of linear associations and deviation from linearity were computed. The first was obtained from a model in which AMH quintiles were entered as a continuous variable and the latter from comparing the linear model to a model in which AMH quintiles were included as four indicator variables using a likelihood ratio test.

A series of multivariable linear regression models were used to examine associations of AMH with each outcome. The crude association is estimated in Model 1. We then adjusted for age, pubertal stage, age at menarche, ethnicity, socioeconomic position, fat mass, height, height-squared, and hormonal contraceptive use (Model 2). The inclusion of height and height-squared as covariables in the analyses that included fat mass was to ensure adjustment for greater relative adiposity, rather than greater fat mass as a result of greater height. To make coefficients from multivariable regression models more interpretable, they were multiplied by log base 2 so that results are the mean difference in outcome per doubling of AMH. For outcomes that were logged (fasting insulin, triglycerides and CRP) regression coefficients were back transformed (exponentiated) so that coefficients represent the percentage change in the outcome per doubling of AMH. Analyses were conducted on all participants in our study sample including those taking the hormonal contraceptive pill, with hormonal contraceptive use being adjusted for in the regression model (n = 1,308). Analyses were then repeated after excluding the 170 (13%) participants taking the hormonal contraceptive pill.

### Sensitivity Analyses

Smoking, physical activity and age at menarche were considered as potential confounders. However, there were variable amounts of missing data for each of these confounder; for smoking data was available for 1,096 [83.8%] of those with complete data on all other variables; physical activity data available for 607 [46.4%]; and age at menarche data for 1,191 [91.1%]. To assess potential confounding by these variables we completed sensitivity analyses on the sub-samples for who these data were available. We compared models 1 and 2 in these subsets with equivalent results from the larger main sample of 1,308. We then adjusted for smoking, physical activity and age at menarche in a group of final models (Model 3a, 3b and 3c, respectively). To ensure that females with exceptionally high AMH values were not driving any of the observed associations, we repeated all analyses excluding those females within the highest 3% of the AMH distribution (i.e. over 2 standard deviations above the mean AMH level, N = 63). We also repeated analyses after excluding the 6 (0.46%) participants who had not yet started menarche, as this small group could potentially be experiencing delayed puberty.

## Results

**Table 1** summarises the characteristics of our analysis sample (n = 1,308) and also of those female participants who were eligible but were excluded from the analysis due to missing data for potential confounders or cardiometabolic outcomes. Excluded participants had, on average, higher insulin, glucose and LDLc than those who were included. Participants who were excluded were also more likely to be older, have a later age at menarche, of non-white ethnicity, taking hormonal contraceptives, and less likely to be post-pubertal. However, for most of these characteristics the magnitude of the differences was small.

AMH values ranged from 0.16–35.84 ng/ml and median AMH was 3.57 ng/ml (IQR: 2.41, 5.49). For females classified as post-pubertal, median (IQR) AMH was 3.81 ng/ml (2.55, 5.82) compared with 3.25 ng/ml (2.23, 5.05) in those classed as in early puberty (P value from a Mann-Whitney U-test of the difference between median AMH levels in the two puberty categories ≤0.001).

**Table 2** shows the distributions of cardiometabolic outcomes and confounders by quintiles of AMH. There was some evidence of an inverse linear association of AMH with CRP and age at menarche, and that the association with DXA determined fat mass deviated from linearity; however, BMI and percentages of overweight and obese did not differ relative to quintiles of AMH. There was no strong evidence that the distribution of other outcomes or confounders varied across quintiles of AMH.

**Table 2:** Characteristics of females by quintiles of AMH (n = 1,308).

	Quintiles of AMH						
Variable	1 (0.16–2.14 ng/ml) N = 262	2 (2.15–3.12 ng/ml) N = 262	3 (3.13–4.17 ng/ml) N = 261	4 (4.18–6.19 ng/ml) N = 262	5 (6.20–35.84 ng/ml) N = 261	Linear P-value	Deviation from linearity P value
**Outcomes**							
Median Insulin-iu/l (IQR)*	10.29 (7.44, 13.16)	9.39 (7.39, 12.43)	9.14 (7.27, 12.73)	10.07 (7.53, 13.21)	9.84 (7.23, 12.39)	0.99	0.07
**Mean Glucose-mmol/l (SD)**	5.13 (0.37)	5.11 0.35)	5.14 (0.30)	5.10 (0.36)	5.13 (0.32)	0.99	0.50
**Mean HDLc-mmol/l (SD)**	1.35 (0.30)	1.38 (0.32)	1.36 (0.29)	1.33 (0.29)	1.35 (0.30)	0.37	0.46
**Mean LDLc-mmol/l (SD)**	2.13 (0.59)	2.17 (0.55)	2.18 (0.56)	2.17 (0.55)	2.17 (0.55)	0.50	0.91
Median Trigs-mmol/l (IQR)*	0.81 (0.65, 1.01)	0.73 (0.60, 1.00)	0.75 (0.61, 0.95)	0.76 (0.63, 1.00)	0.78 (0.61, 0.95)	0.36	0.19
Median CRP-mg/l (IQR)*	0.43 (0.23, 1.02)	0.41 (0.23, 1.02)	0.37 (0.21, 0.81)	0.34 (0.22, 0.63)	0.39 (0.20, 0.92)	0.02	0.13
**Potential confounders**							
**Mean age in years (SD)**	15.42 (0.29)	15.40 (0.22)	15.42 (0.27)	15.42 (0.28)	15.43 (0.28)	0.44	0.93
**Median age at menarche in years (IQR) n = 1191**	13.00 (12.00, 14.00)	12.83 (12.00, 13.75)	12.92 (11.92, 13.58)	12.67 (11.83, 13.50)	12.83 (11.75, 13.58)	<0.01	0.48
**Mean birthweight in kg (SD)**	3.40 (0.50)	3.35 (0.45)	3.37 (0.50)	3.39 (0.49)	3.38 (0.47)	0.86	0.59
**Mean gestational age in weeks (SD)**	39.61 (1.70)	39.52 (1.63)	39.52 (1.78)	39.63 (1.57)	39.70 (1.63)	0.35	0.70
**Fat mass z-score (SD)**	0.03 (1.00)	−0.08 (0.86)	0.07 (1.00)	0.12 (0.96)	−0.02 (0.98)	0.59	0.09
**Mean BMI (SD)**	21.92 (3.62)	21.58 (3.26)	21.98 (3.52)	22.16 (3.50)	21.74 (3.83)	0.75	0.26
**Body Mass Index (n)**						0.65	0.19
Underweight	8.0% (21)	7.2% (19)	3.5% (9)	5.7% (15)	6.9% (18)		
Normal weight	69.9% (183)	77.1% (202)	74.3% (194)	70.6% (185)	74.0% (193)		
Overweight	18.7% (49)	13.0% (34)	17.2% (45)	19.5% (51)	12.6% (33)		
Obese	3.4% (9)	2.7% (7)	5.0% (13)	4.2 (11)	6.5% (17)		
**SEP of head of household (n)**							
Non-manual worker	88.9% (233)	85.1% (223)	86.2% (225)	86.3% (226)	84.3% (220)	0.23	0.74
**Ethnicity (n)**							
Non-white	3.1% (8)	4.6% (12)	2.7% (7)	3.4% (9)	3.5% (9)	0.92	0.66
**Using hormonal contraceptives**							
Yes	17.2% (45)	13.7% (36)	9.2% (24)	13.4% (35)	11.5% (30)	0.07	0.18
**Covariables additionally adjusted for in the sensitivity analyses**							
**Physical Activity (n) = 607**							
Median CPM in 1 week (IQR)	433.32 (316.71, 516.97)	423.07 (348.26, 565.23)	412.13 (350.90, 503.82)	416.97 (334.21, 492.67)	422.84 (314.83, 504.98)	0.37	0.59
Median No. of mins in MVPA (IQR)	13.67 (6.50, 25.40)	16.75 (8.33, 27.40)	15.67 (10.00, 27.00)	15.00 (7.17, 22.25)	14.86 (6.80, 28.67)	0.63	0.67
**Ever smoked (n) n = 1096**							
Yes	33.6% (75)	32.9% (72)	28.8% (63)	24.8% (54)	31.7% (69)	0.22	0.28
**Frequency of smoking (n) n = 1096**							
1+ per week	1.8% (4)	3.7% (8)	3.2% (7)	3.2% (7)	1.4% (3)	0.28	0.51

SD - standard deviation. CPM – counts per minute. Trigs - triglyceride Linear P values are from a regression in which quintiles of AMH were entered as a continuous variable. Deviation from linearity P values are from a likelihood ratio test in which results from the above regression model are compared to those from a model with quintiles of AMH entered as an indicator variable, and a small p-value should be interpreted as evidence against the null hypothesis that the relationship is linear. *P values are from a regression of log transformed variables.

Multivariable associations of AMH with all outcomes are displayed in **Table 3**. After adjustment for confounders, AMH was not associated with any of the cardiometabolic outcomes. Results did not change substantially after the 170 (13%) participants taking hormonal contraceptives were removed from the analyses (Table 4). The percentage change coefficient for the association between AMH and CRP changed direction (−3%, 95%CI −8% to+2% in the analysis including those that were taking hormonal contraceptives compared to+1%, 95% CI −5% to+7% when participants taking hormonal contraceptives were excluded). However, neither of these associations reached the conventional 5% significance level.

**Table 3:** Multivariable associations of AMH with cardiometabolic risk factors (n = 1,308).

	Model 1			Model 2		
	Coeff	95% CI	P	Coeff	95% CI	P
	Mean difference per doubling of AMH					
**Glucose mmol/l**	−0.003	−0.02, 0. 02	0.73	−0.004	−0.02, 0.01	0.64
**HDL-c mmol/l**	−0.004	−0.02, 0.01	0.63	−0.006	−0.02, 0.009	0.42
**LDL-c mmol/l**	0.004	−0.03, 0.03	0.79	0.01	−0.02, 0.04	0.43
	Ratio of geometric means per doubling of AMH					
**Insulin iu/l**	−1%	−3%,+2%	0.56	−1%	−3%,+2%	0.56
**Triglyceride mmol/l**	0%	−2%,+2%	0.74	0%	−2%,+2%	0.94
**CRP mg/l**	−4%	−9%,+2%	0.19	−3%	−8%,+2%	0.22

Model 1– crude estimate.

Model 2– additionally adjusted for birth weight, gestational age, pubertal stage, age, age at menarche, ethnicity, socioeconomic position, fat mass, height and height^2^ and use of the hormonal contraceptive pill.

**Table 4:** Multivariable associations of AMH with cardiometabolic risk after excluding 170 (13%) participants taking the hormonal contraceptive pill (n = 1,138).

	Model 1			Model 2		
	Coeff	95% CI	P	Coeff	95% CI	P
	Mean difference per doubling of AMH					
**Glucose mmol/l**	−0.01	−0.03, 0.007	0.19	−0.01	−0.03, 0.008	0.23
**HDL-c mmol/l**	−0.003,	−0.02, 0.01	0.72	−0.004	−0.02, 0.01	0.65
**LDL-c mmol/l**	0.006	−0.03, 0.04	0.71	0.01	−0.02, 0.01	0.52
	Ratio of geometric means per doubling of AMH					
**Insulin iu/l**	−1%	−3%,+2%	0.63	−1%	−3%,+2%	0.53
**Triglyceride mmol/l**	0%	−2%,+2%	0.99	0%	−2%,+2%	0.85
**CRP mg/l**	+2%	−4%,+8%	0.61	+1%	−5%,+7%	0.73

Model 1– crude estimate.

Model 2– additionally adjusted for birth weight, gestational age, pubertal stage, age, age at menarche, ethnicity, socioeconomic position, fat mass, height and height^2.^

### Sensitivity Analyses

**Table S1–S3** show the multivariable analyses of AMH with cardiometabolic outcomes in the subgroup of participants who additionally had complete data on smoking, physical activity and age at menarche, respectively. Results did not differ substantially in the subgroups with smoking, physical activity and age at menarche data to those in the main analysis shown in **Table 2**. This suggests that missing data for these potential confounding factors did not result in selection bias. Additional adjustment for smoking, physical activity or age at menarche did not alter results. Results were largely unchanged when females in the highest 3% of the AMH distribution were excluded (**Table S4**) or when those that had not started menarche were excluded (**Table S5**).

## Discussion

In this study we demonstrated that AMH was not associated with a range of cardiometabolic risk factors including lipids and insulin in a large general population of adolescent females. In accordance with recent longitudinal and cross-sectional studies, [32], [33], [49] we identified that AMH levels were slightly higher in females assessed post-puberty, compared with those assessed during early puberty, however adjusting for pubertal status did not alter the lack of association of AMH with cardiometabolic risk factors.

Although we have modelled AMH as the exposure and the cardiometabolic risk factors as the outcomes, given that they are measured at the same time their respective classification as exposure and outcome are interchangeable. Whilst studies of this association to date (see introduction) have been interpreted as examining the hypothesis that more rapid ovarian ageing (that AMH proxies for) may be associated with more adverse cardiometabolic outcomes, the opposite has also been proposed. Hyperinsulinaemia has been proposed as a possible mechanism that may disrupt normal follicular development, [50] with polycystic ovary syndrome associated with both hyperinsulinaemia and marked increases in AMH, [20], [25] however in the current study we did not see an association between AMH and insulin, and there was also no evidence of a non-linear association suggestive of a threshold effect.

We are aware of only one other study that has examined the association of AMH with CRP, in adult Asian women (n  = 290). [51] In that study of a mixed population of women with and without PCOS, consistent with our own findings, no overall association across the distributions of both AMH and CRP was observed. Lin et al reported AMH to be positively associated with total cholesterol, both HDL-C and LDL-C in a mixed population of women with and without PCOS, but this finding was not replicated in an analysis of adult ovulatory women. [52] Furthermore in an animal model of atherosclerosis, AMH was not associated with lipid concentrations. [24] Further large studies are required to ascertain whether AMH is associated with an adverse lipid profile in adolescents.

Our null results could be because there is, in fact, no association of AMH with cardiometabolic outcomes, and the contradictory results in select adult women to date represent the expected variation around a true null association. It is also possible that associations observed in previous studies are explained by confounding, either because they did not appropriately adjust for confounders such as socioeconomic position or fat mass, or because they had inadequate measurements of potential confounders. It is notable that in our study of adolescent females, where confounding by characteristics such as smoking or an effect of existing undiagnosed atherosclerosis on AMH is highly unlikely, we did not observe an association even before multivariable adjustment for potential confounders. In previous publications we have shown expected associations with fasting insulin, glucose and the other cardiovascular risk factors examined here, for example we have shown a dose response association of BMI, waist circumference and total body fat mass with these outcomes, [53] illustrating that the null results presented here are highly unlikely to be due to error in the outcome measurements. It is possible that associations of AMH with cardiometabolic outcomes emerge only at a later age and/or exist only in sub-groups of the population such as those with PCOS. Future prospective studies with AMH and cardiovascular risk factors in adult premenopausal women are needed to ascertain whether ovarian reserve is independently associated with cardiovascular risk over the established associations with oestrogen production. Overall our findings raise the possibility that functional ovarian reserve as assessed by AMH is not a direct determinant of the lipid and insulin cardiometabolic risk profile during the early reproductive years in healthy women.

### Strengths and Limitations

To our knowledge, this is the largest study to date to examine associations of AMH with a range cardiometabolic factors in a general adolescent female population. The cross-sectional design does not allow us to establish causality or the direction of association. Some potential confounders (smoking, physical activity and age at menarche) were only available in subgroups of participants, but analyses in these subgroups did not suggest they were importantly different from the main analysis sample or that these characteristics were important confounders. As is common in birth cohort studies there has been loss to follow-up over time (Figure 1) and participants who were excluded from our study because of missing data differed slightly for some characteristics compared to those who were included (Table 1). Our results would only be biased if associations were markedly different in those lost to follow-up or with missing data compared to those included in our analyses, and whilst we cannot test this, we cannot think of any reason as to why this would be the case. Consistent with other large epidemiological studies conducted in general population samples; we are not able to directly measure insulin resistance using the gold standard euglycaemic hyperinsulinemic clamp. Fasting insulin has been shown to have modest to strong correlations with clamp assessed insulin resistance (correlation coefficients 0.5 to 0.9) in children and adolescents. [42] Any measurement error is likely to be non-differential and therefore would be expected to bias results towards the null. However, even if non-differential measurement error resulted in our associations being weaker than they would be with a direct measure of insulin resistance, with correlations of 0.5 to 0.9 we would have expected some association of AMH with fasting insulin if this were an important determinant and/or predictor. For this study we were unable to conduct physical assessments of pubertal development by trained clinical personnel. In the absence of a direct visual assessment by a clinician, self-assessment of Tanner stage is considered a practical and valid choice, particularly for longitudinal studies with repeat assessments. [54] Kappa coefficients comparing clinician to self-assessments range from 0.3 to 0.9 for breast and from 0.4 to 0.9 for pubic-hair development in different studies. [55] Our study used the drawings of pubertal stages developed by Morris and Udry, [46] which had been successfully used in previous cohort studies. [56], [57] We do not have diagnostic information on whether female participants had PCOS; however removing participants with an AMH >2 standard deviations from the mean (the highest 3% of the AMH distribution) did not change any associations. We adopted this approach as the adult diagnostic criteria for PCOS has recently been recognised as not appropriate for adolescent girls, [58] although AMH is elevated in adolescents with polycystic ovarian morphology and in PCOS. [59] We also do not have measures of testosterone and could therefore not investigate the association between androgens and AMH. Lastly our population are largely of European, white ethnic origin and we cannot assume that results would generalise to other ethnicities.

In conclusion, we have found that in a general population of adolescent females, AMH is not associated with fasting insulin or other cardiometabolic outcomes. There is a need for further large prospective studies of these associations that include repeat measurements with increasing age in order to understand whether AMH, a marker of ovarian reserve, is importantly related to cardiometabolic health in healthy women as they age from early adolescence to peak reproductive age and beyond.

## Supporting Information

Table S1 **Multivariable associations of AMH with cardiometabolic outcomes in participants with complete data on all variables including smoking.**
(DOCX)

Table S2 **Multivariable associations of AMH with cardiometabolic outcomes in participants with complete data on all variables including minutes spent in moderate to vigorous activity and counts per minute (n = 607).**
(DOCX)

Table S3 **Multivariable associations of AMH with cardiometabolic outcomes in participants with complete data on all variables including age at menarche (n = 1,191).**
(DOCX)

Table S4 **Multivariable associations of AMH with cardiometabolic risk factors, excluding females with the top 3% of AMH values.**
(DOCX)

Table S5 **Multivariable associations of AMH with cardiometabolic risk factors, excluding those that have not yet started.**
(DOCX)
