# Supplementary material for: Anti-Müllerian Hormone Is Not Associated with Cardiometabolic Risk Factors in Adolescent Females
Source: PLoS One. 2013 May 31;8(5):e64510. doi: 10.1371/journal.pone.0064510 (PMC3675909; doi:10.1371/journal.pone.0064510)
Supplement: Table S1 — Multivariable associations of AMH with cardiometabolic outcomes in participants with complete data on all variables including smoking. (DOCX) [file pone.0064510.s001.docx]

**Electronic Appendices –**

**S1 – Multivariable associations of AMH with cardiometabolic outcomes in participants with complete data on all variables including smoking (n=1,096)**

|  | Model 1 | | |  | | Model 2 | | |  | Model 3a | | |
| --- | --- | --- | --- | --- | --- | --- | --- | --- | --- | --- | --- | --- |
|  | Coeff | 95% CI | P |  | | Coeff | 95% CI | P |  | Coeff | 95% CI | P |
|  | Mean difference per doubling of AMH | | | | | | | | | | | |
| **Glucose mmol/l** | -0.007 | -0.03, 0.01 | 0.48 |  | -0.008 | | -0.03, 0.01 | 0.42 |  | -0.009 | -0.03, 0.01 | 0.40 |
|  |  |  |  |  |  | |  |  |  |  |  |  |
| **HDL-c mmol/l** | -0.007 | -0.02, 0.01 | 0.45 |  | -0.009 | | -0.03, 0.008 | 0.30 |  | -0.009 | -0.03, 0.008 | 0.31 |
|  |  |  |  |  |  | |  |  |  |  |  |  |
| **LDL-c mmol/l** | 0.0003 | -0.03, 0.03 | 0.99 |  | 0.009 | | -0.02, 0.04 | 0.58 |  | 0.009 | -0.02, 0.04 | 0.60 |
|  | Percentage change per doubling of AMH | | | | | | | | | | | |
| **Insulin iu/l** | 0% | -3%, +2% | 0.77 |  | 0% | | -3%, +2% | 0.87 |  | 0% | -3%, +2% | 0.86 |
|  |  |  |  |  |  | |  |  |  |  |  |  |
| **Triglyceride mmol/l** | -1% | -3%, +2% | 0.41 |  | 0% | | -3%, +2% | 0.65 |  | 0% | -3%, +2% | 0.66 |
|  |  |  |  |  |  | |  |  |  |  |  |  |
| **CRP mg/l** | -4% | -10%, +2% | 0.20 |  | -3% | | -8%, +2% | 0.30 |  | -3% | -8%, +3% | 0.31 |
