# Supplementary material for: Anti-Müllerian Hormone Is Not Associated with Cardiometabolic Risk Factors in Adolescent Females
Source: PLoS One. 2013 May 31;8(5):e64510. doi: 10.1371/journal.pone.0064510 (PMC3675909; doi:10.1371/journal.pone.0064510)
Supplement: Table S2 — Multivariable associations of AMH with cardiometabolic outcomes in participants with complete data on all variables including minutes spent in moderate to vigorous activity and counts per minute (n = 607). (DOCX) [file pone.0064510.s002.docx]

**S2 – Multivariable associations of AMH with cardiometabolic outcomes in participants with complete data on all variables including minutes spent in moderate to vigorous activity and counts per minute (n=607)**

|  | Model 1 | | |  | | Model 2 | | |  | Model 3a | | |
| --- | --- | --- | --- | --- | --- | --- | --- | --- | --- | --- | --- | --- |
|  | Coeff | 95% CI | P |  | | Coeff | 95% CI | P |  | Coeff | 95% CI | P |
|  | Mean difference per doubling of AMH | | | | | | | | | | | |
| **Glucose mmol/l** | 0.003 | -0.02, 0.03 | 0.81 |  | 0.003 | | -0.03, 1.03 | 0.86 |  | 0.001 | -0.03, 0.03 | 0.94 |
|  |  |  |  |  |  | |  |  |  |  |  |  |
| **HDL-c mmol/l** | -0.002 | -0.03, 0.02 | 0.89 |  | -0.005 | | -0.03, 0.02 | 0.68 |  | -0.006 | -0.03, 0.02 | 0.63 |
|  |  |  |  |  |  | |  |  |  |  |  |  |
| **LDL-c mmol/l** | 0.01 | -0.03, 0.06 | 0.63 |  | 0.02 | | -0.0, 0.07 | 0.39 |  | 0.02 | -0.02, 0.07 | 0.36 |
|  | Percentage change per doubling of AMH | | | | | | | | | | | |
| **Insulin iu/l** | -2% | -5%, +2% | 0.32 |  | -1% | | -5%, +2% | 0.37 |  | -1% | -3%, +2% | 0.41 |
|  |  |  |  |  |  | |  |  |  |  |  |  |
| **Triglyceride mmol/l** | -1% | -4%, +2% | 0.45 |  | -1% | | -4%, +2% | 0.68 |  | -1% | -3%, +2% | 0.72 |
|  |  |  |  |  |  | |  |  |  |  |  |  |
| **CRP mg/l** | -10% | -17%, -3% | <0.01 |  | -9% | | -16%, -2% | 1.01 |  | -9% | -22%, -2% | 0.02 |
