# Supplementary material for: Anti-Müllerian Hormone Is Not Associated with Cardiometabolic Risk Factors in Adolescent Females
Source: PLoS One. 2013 May 31;8(5):e64510. doi: 10.1371/journal.pone.0064510 (PMC3675909; doi:10.1371/journal.pone.0064510)
Supplement: Table S3 — Multivariable associations of AMH with cardiometabolic outcomes in participants with complete data on all variables including age at menarche (n = 1,191). (DOCX) [file pone.0064510.s003.docx]

**S3 – Multivariable associations of AMH with cardiometabolic outcomes in participants with complete data on all variables including age at menarche (n=1,191)**

|  | Model 1 | | |  | | Model 2 | | |  | Model 3a | | |
| --- | --- | --- | --- | --- | --- | --- | --- | --- | --- | --- | --- | --- |
|  | Coeff | 95% CI | P |  | | Coeff | 95% CI | P |  | Coeff | 95% CI | P |
|  | Mean difference per doubling of AMH | | | | | | | | | | | |
| **Glucose mmol/l** | -0.006 | -0.03, 0.01 | 0.52 |  | -0.008 | | -0.03, 0.01 | 0.44 |  | -0.005 | -0.02, 0.01 | 0.61 |
|  |  |  |  |  |  | |  |  |  |  |  |  |
| **HDL-c mmol/l** | -0.01 | -0.03, 0.005 | 0.17 |  | -0.01 | | -0.03, 0.003 | 0.11 |  | -0.01 | -0.03, 0.004 | 0.13 |
|  |  |  |  |  |  | |  |  |  |  |  |  |
| **LDL-c mmol/l** | 0.01 | -0.02, 0.04 | 0.55 |  | 0.02 | | -0.02, 0.05 | 0.32 |  | 0.02 | -0.01, 0.05 | 0.19 |
|  | Percentage change per doubling of AMH | | | | | | | | | | | |
| **Insulin iu/l** | -1% | -3%, +2% | 0.55 |  | -1% | | -3%, +2% | 0.49 |  | -1% | -3%, +2% | 0.60 |
|  |  |  |  |  |  | |  |  |  |  |  |  |
| **Triglyceride mmol/l** | 0% | -2%, +2% | 0.88 |  | 0% | | -2%, +2% | 0.69 |  | +1% | -1%, +3% | 0.47 |
|  |  |  |  |  |  | |  |  |  |  |  |  |
| **CRP mg/l** | -2% | -8%, +4% | 0.49 |  | -2% | | -7%, +4% | 0.48 |  | 3% | -8%, 3% | 0.36 |
