# Supplementary material for: Anti-Müllerian Hormone Is Not Associated with Cardiometabolic Risk Factors in Adolescent Females
Source: PLoS One. 2013 May 31;8(5):e64510. doi: 10.1371/journal.pone.0064510 (PMC3675909; doi:10.1371/journal.pone.0064510)
Supplement: Table S4 — Multivariable associations of AMH with cardiometabolic risk factors, excluding females with the top 3% of AMH values. (DOCX) [file pone.0064510.s004.docx]

**S4: Multivariable associations of AMH with cardiometabolic risk factors, excluding females with the top 3% of AMH values (n=1,245)**

|  | Model 1 | | |  | Model 2 | | |
| --- | --- | --- | --- | --- | --- | --- | --- |
|  | Coeff | 95% CI | P |  | Coeff | 95% CI | P |
|  | Mean difference per doubling of AMH | | | | | | |
| **Glucose mmol/l** | -0.004 | -0.02, 0.02 | 0.73 |  | -0.005 | -0.03, 0.02 | 0.67 |
|  |  |  |  |  |  |  |  |
| **HDL-c mmol/l** | 0.0001 | -0.02, 0.02 | 0.99 |  | -.0009 | -0.02 0.02 | 0.92 |
|  |  |  |  |  |  |  |  |
| **LDL-c mmol/l** | 0.008 | -0.03, 0.04 | 0.65 |  | 0.02 | -0.02, 0.05 | 0.38 |
|  | Percentage change per doubling of AMH | | | | | | |
| **Insulin iu/l** | 0% | -3%, +2% | 0.88 |  | 0% | -3%, +2% | 0.82 |
|  |  |  |  |  |  |  |  |
| **Triglyceride mmol/l** | -2% | -4%, +1% | 0.18 |  | -1% | -3%, +3% | 0.31 |
|  |  |  |  |  |  |  |  |
| **CRP mg/l** | -3% | -10%, +3% | 0.29 |  | -3% | -9%, +3% | 0.28 |
