# Supplementary material for: Anti-Müllerian Hormone Is Not Associated with Cardiometabolic Risk Factors in Adolescent Females
Source: PLoS One. 2013 May 31;8(5):e64510. doi: 10.1371/journal.pone.0064510 (PMC3675909; doi:10.1371/journal.pone.0064510)
Supplement: Table S5 — Multivariable associations of AMH with cardiometabolic risk factors, excluding those that have not yet started. (DOCX) [file pone.0064510.s005.docx]

**S5: Multivariable associations of AMH with cardiometabolic risk factors, excluding those that have not yet started menarche (n=1, 302)**

|  | Model 1 | | |  | Model 2 | | |
| --- | --- | --- | --- | --- | --- | --- | --- |
|  | Coeff | 95% CI | P |  | Coeff | 95% CI | P |
|  | Mean difference per doubling of AMH | | | | | | |
| **Glucose mmol/l** | -0.003 | -0.02, 0.02 | 0.73 |  | -.004 | -0.02, 0.01 | 0.66 |
|  |  |  |  |  |  |  |  |
| **HDL-c mmol/l** | -0.004 | -0.02, 0.02 | 0.66 |  | -0.007 | -0.02, 0.009 | 0.40 |
|  |  |  |  |  |  |  |  |
| **LDL-c mmol/l** | -0.004 | -0.03, 0.03 | 0.80 |  | -0.007 | -0.02, 0.04 | 0.46 |
|  | Percentage change per doubling of AMH | | | | | | |
| **Insulin iu/l** | -1% | -3%, +2% | 0.63 |  | 0% | -3%, +2% | 0.66 |
|  |  |  |  |  |  |  |  |
| **Triglyceride mmol/l** | 0% | -2%, +2% | 0.79 |  | 0% | -2%, +2% | 0.91 |
|  |  |  |  |  |  |  |  |
| **CRP mg/l** | -4% | -10%, +2% | 0.16 |  | -3% | -9%, +2% | 0.19 |
